# On Tubal Moles

**Published:** 1908-12-05

**Authors:** 


### Obstetrics.

### ON TUBAL MOLES.

The formation of a mole in the Fallopian tube, that is to say the conversion of a tubal gestation into a mass resembling a common carneous mole, is a not infrequent termination of an ectopic gestation. It is a far commoner occurrence than rupture of the tube, and it is quite possible that it happens more frequently even than we know of. It is only in the cases which end by extrusion of the mole from the abdominal ostium of the tube accompanied by peritoneal hæmorrhage (tubal abortion) that the condition is evident, though moles are occasionally discovered when an operation is undertaken for "unruptured tubal gestation." The knowledge acquired in recent years of the pathological anatomy of ectopic gestation, and of the method of embedding of the fertilised ovum in the uterus as well as in the tube, has done much to clear up the difficult points in connection with the fate of a gestation sac in the Fallopian tube. Formerly rupture of the tube was looked upon as the usual fate of a tubal gestation, but now we know that this is a comparatively infrequent ending, and that mole formation, with or without extrusion from the abdominal ostium, is the commonest termination.

To understand this conversion of a tubal gestation sac into a mole we must have a clear understanding of its anatomical relations to the tube. Recent research has shown that the fertilised ovum embeds itself in the wall of the tube in exactly the same manner as in that of the uterus. The early embryo is covered by a sheet of protoplasm, full of nuclei, but devoid of cell divisions, which is known as trophoblast; and having what may be called "phagocytic" powers, the trophoblast eats away the tissues in contact with it. Thus the embryo bores its way into the actual wall of the tube, beneath whose epithelium there is a very thin layer of connective tissue, so that it soon reaches the muscular coats. During this process the embryo and its coverings enlarge, so that the chorionic sac is soon larger than the hole through which it entered the tube wall. Thus the margins of this hole become expanded over the chorionic sac, and a kind of reflexa, now known as the "capsularis" is formed. This capsularis may contain muscle fibres, showing that the embryo and chorion have entered the muscle layers. During this embedding process blood vessels are of necessity encountered, and in general, the eating away of their walls gives rise to the formation of small "blood islands" in the trophoblast itself, completely surrounded by trophoblast, and forming the earliest evidence of a maternal blood sinus, into which the villi afterwards dip. If this process of eating away the wall of the tube goes on sufficiently long, it must of necessity happen that the tube wall is at last quite destroyed on one side, and consequently rupture occurs and hæmorrhage follows. But before this happens, in the majority of cases the opening up of blood vessels gives rise to hæmorrhage, not only into the trophoblast, but outside its area into the potential space between the advancing trophoblast and the tube wall. This blood coagulates around the villi of the trophoblast, compresses the small amniotic sac and eventually forms an almost solid mass, whose structure is essentially similar to that of a uterine carneous mole.

The great and sudden accession of bulk thus produced in the tube leads to distension and consequent pressure on nerve endings, and is the cause of the premonitory pains in the pelvis which are classical and which herald the final end of the condition. It is this pain together with an unusual lump in the pelvis, with perhaps one missed menstrual period which make up the clinical picture and provide grounds for operations in certain cases. We know very little about the fate of tubal moles which remain *in situ* and do not cause peritoneal hæmorrhage. It is highly probable, however, not only that they do occur, but also that after a period of pelvic discomfort they are slowly absorbed, with complete restoration of the tube lumen. The more common fate of a tubal mole, however, is extrusion from the abdominal ostium, or hæmorrhage into the peritoneum through the lumen of the tube and formation of a hæmatocele without actual extrusion of the gestation sac. In cases when the gestation sac is situated in the ampullary end of the tube, the gradual enlargement of necessity opens up the abdominal ostium. Sooner or later the gestation sac must protrude through the ostium, and when the greatest diameter is past the ostium the tube wall retracts by muscular action or elasticity, and the embryo and its coverings are extruded (tubal abortion). This means separation from its attachments and consequent hæmorrhage. The amount of peritoneal hæmorrhage in these cases is not nearly so great as in tubal ruptures, and being more slowly poured out forms a localised hæmatocele around the abdominal ostium (peritubal hæmatocele). This is the condition so commonly found upon opening the abdomen in suspected cases. The dénouement is not dramatic in all cases, as in tubal rupture, but may occur so gradually as to give very few symptoms. The condition then is only diagnosed after several days or weeks when adhesions have formed, and the hæmatocele begins to cause pain by pressure and traction, accompanied as a rule by prolonged uterine bleeding. In other cases partial separation of the mole from its attachments cause hæmorrhage which may at once find its way into the tube lumen, and so to the peritoneum, or may track along the muscle layers until it reaches the peritoneum. In this way again a peritubal hæmatocele may be formed if the blood issues from the abnominal ostium, or a paratubal hæmatocele if the blood tracks along and perforates the peritoneal coat. It is possible that an embryo may not be wholly separated from its attachments by these accidents, and as a result may be just sufficiently nourished to go on growing. In this case further hæmorrhage may occur at a later date, or the embryo, by extending its area of attachment may even go on to an advanced period of pregnancy.

